# Body Weight, Length and Head Circumference at Birth in a Cohort of Turkish Newborns

**DOI:** 10.4274/Jcrpe.693

**Published:** 2012-09-11

**Authors:** Selim Kurtoğlu, Nihal Hatipoğlu, Mustafa Mümtaz Mazıcıoğlu, Mustafa Ali Akın, Dilek Çoban, Sonay Gökoğlu, Osman Baştuğ

**Affiliations:** 1 Erciyes University Medical Faculty, Department of Pediatric Endocrinology, Kayseri, Turkey; 2 Erciyes University Medical Faculty, Department of Family Medicine, Kayseri, Turkey; 3 Erciyes University Medical Faculty, Department of Neonatology, Kayseri, Turkey; 4 Kayseri Maternity and Child Hospital, Department of Pediatric Development, Kayseri, Turkey

**Keywords:** Intrauterine growth percentiles, SGA, LGA

## Abstract

**Objective:** Intrauterine growth references are primarily useful indicators in the assessment of the general health status of newborn infants. Although Lubchenco’s references are still used in many neonatal care units, we believe that there is a need for up-to-date intrauterine growth references specific for different populations. To develop gestational age-and gender-specific national references for birth weight, birth length and head circumference.

**Methods:** Data were collected from neonatal records of perinatology services of eleven hospitals from January to December 2009. The anthropometry of a total of 4750 singleton live births born between 28 and 41 weeks of gestation were recorded. Means and standard deviations were calculated, and percentiles for each gender and gestational week were produced using the LMS program. The results were compared with US infants and also with local data.

**Results:** Gestational age- and gender-specific 3^rd^, 5^th^, 10^th^, 15^th^, 25^th^, 50^th^, 75^th^, 85^th^, 90^th^, 95^th^ and 97^th^ percentile values were produced. Comparison of the 10^th^, 50^th^ and 90^th^ percentile values showed that the boys were heavier and longer than the girls. Head circumference values were also higher in the boys. Proportions of small for gestational age (SGA), appropriate for gestational age (AGA) and large for gestational age (LGA) infants in the sample were 10.1%, 79.1% and 10.8%, respectively.

**Conclusion:** These gender- and gestational age-specific references will be of use in clinical practice and also for research purposes until more comprehensive, reliable and accessible national data pertaining to the intrauterine growth of Turkish infants are produced.

**Conflict of interest:**None declared.

## INTRODUCTION

Body weight, length and head circumference at birth are measurements used in the assessment of peri- and postnatal growth and health. However, for evaluation of fetal growth, it is essential to know the gestational age-adjusted birth weight, length and head circumference (1). It is known that there is a significant relationship between preterm birth and neonatal complications, mortality and developmental delay (2). The abnormal birth weight of infants who are small for gestational age (SGA) or of those who are large for gestational age (LGA) is also related with birth complications as well as with an increased risk of cardiovascular disease, obesity and hypertension in later life (3,4,5). 

Producing gestational age-specific percentiles provides the opportunity to determine SGA and LGA infants. Another application of intrauterine percentiles is in predicting whether a preterm infant will be able to maintain his/her growth velocity or achieve catch-up growth in postnatal life (6). In 1966, Lubchenco et al (7) produced the first intrauterine growth reference charts which were based on a small sample of newborns whose mothers’ socioeconomic level was low. These were the first and most frequently used reference charts until 1976, when another set of reference charts based on a relatively large sample size was published (8). Neither of these references may be representative of current populations.

It is known that fetal, maternal, placental and environmental factors may all influence fetal growth (9). Geographic location also plays a significant role. Thus, intrauterine growth reference values, as is also true for other anthropometric measurements, are variable in different populations and regions even in the same country (10,11,12). 

In Turkey, there are a few studies on intrauterine growth, which were conducted in single centers or with small sample sizes. Another limitation of these studies is that either they are based on a single parameter like birth weight or they are not detailed enough to get gestational age-specific percentiles (13,14,15,16,17).

This study was designed to produce gestational age- and gender-adjusted percentile charts in a cohort of Turkish newborns born in a city in the Central Anatolian Region of Turkey. These references will also serve to determine the prevalence of appropriate, SGA and LGA newborns in the community.

## METHODS

This is a cross-sectional and retrospective study in which data were collected from the medical records of infants born between January and December 2009 in 11 hospitals in Kayseri, a Central Anatolian city in Turkey. 65% of the deliveries had taken place in private, 26% in state and 9% in University hospitals. Gestational ages were recorded by the obstetricians or trained nurses in labor wards. A total of 5421 infants born at gestational ages between 28-42 weeks were recruited. Since the numbers of infants between 28-33 and 41-42 weeks of gestation were too small, we combined these age groups as 28-29, 30-31, 32-33 and 41-42 weeks. We excluded infants whose mothers had chronic diseases, who were smokers or who had undergone multiple deliveries. Infants who had fetal health problems, congenital malformations and those with missing data for measurements of weight, length or head circumference were also excluded. To get a smooth distribution for weight, length and head circumference percentiles, we also removed the original data less than 3^rd^ percentiles or higher than the 97^th^ percentiles. Finally, we used the data from 4750 infants to produce our charts.

Preterm delivery was defined as birth before completion of 38 weeks of gestation. SGA was defined as a low measurement than normal according to gestational age and gender. The 10^th^ percentile and 90^th ^percentile cut-off values were used to define SGA and LGA. 

**Statistical Analysis**

Descriptive statistics for each gestational age within sex were performed using the SPSS version 15.0 (Chicago, IL, USA). 

To construct the gestational age- and gender-specific 3^rd^, 10^th^, 15^th^, 25^th^, 50^th^, 75^th^, 85^th^, 90^th^ and 97^th^ percentile curves for birth weight, length and head circumference, we used the LMS Chart Maker Pro version 2.3 software program (The Institute of Child Health, London), which fits smooth centile curves to reference data. This method summarizes percentiles at each gestational age based on the power of age-specific Box-Cox power transformations that are used to normalize data. These three quantities depend on gestational age. The final curves of percentiles are produced by three smooth curves representing L (lambda, skewness), M (mu, median) and S (sigma, coefficient of variation). The calculated 10^th^, 50^th^, and 90^th^ percentiles were compared with the available data on USA infants since both studies were conducted in similar periods. To make local comparisons, we used the most recent study in which 3^rd^, 10^th^, 50^th^, 90^th^, 97^th^ percentiles for term infants of both genders were reported. 

## RESULTS

There were 2493 (52.5%) male and 2257 (47.5%) female infants included in the study. 39.4% of the infants were preterm and 60.6% were term newborns. Gestational age- and gender-specific descriptive values including birth weight, length and head circumference are shown in Table 1. The gestational age- and gender-specific 3^rd^, 5^th^, 10^th^, 15^th^, 25^th^, 50^th^, 75^th^, 85^th^, 90^th^, 95^th^ and 97^th^ percentile values for all parameters are shown in Tables 2,3, 4, 5, 6, 7.

**Figure 1:**
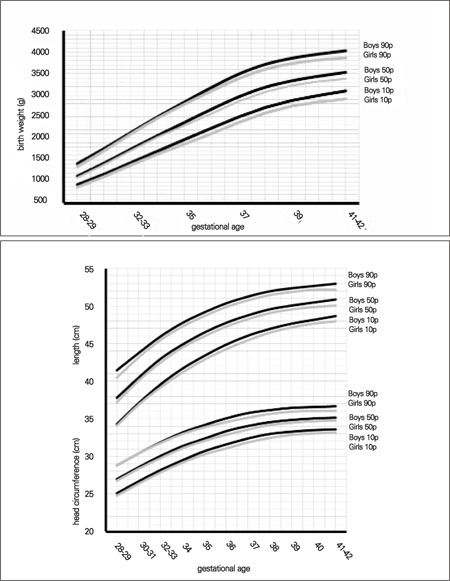
Comparison of 10th, 50th and 90th centiles in male and female neonates for birth weight, birth length and head circumference at birth

Comparison of the 10^th^, 50^th^ and 90^th^ percentiles between genders showed that boys were heavier and longer than girls. Head circumference measurements were also higher in the boys. The largest differences between male and female infants at birth were less than 200 g for weight, 0.8 cm for length and 0.6 cm for head circumference (Figure 1). 

The distribution of SGA, appropriate for gestational age (AGA) and LGA was 10.1%, 79.1% and 10.8%, respectively. 

**Figure 2:**
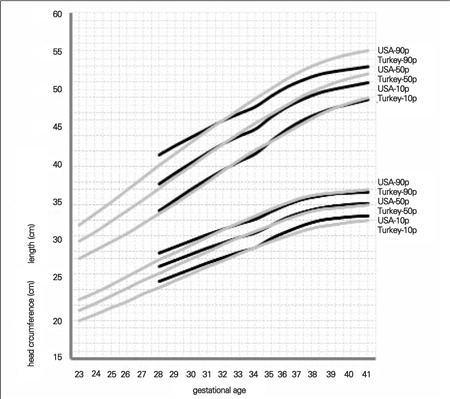
Comparison of 10th, 50th and 90th centiles in Turkish and US newborns for birth weight, birth length and head circumference at birth (boys)

**Figure 3:**
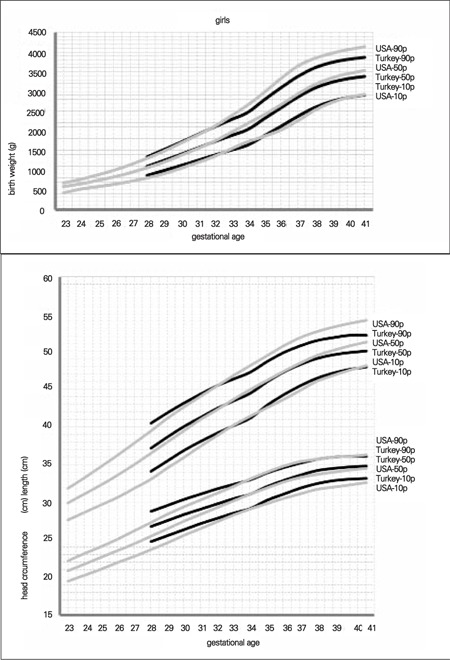
Comparison of 10th, 50th and 90th centiles in Turkish and US newborns for birth weight, birth length and head circumference at birth (girls)

Since there were no comparable data available from local studies, we compared our data with the 10^th^, 50^th^ and 90^th^ percentile values reported for USA infants in a recent study conducted on infants born after 32 weeks of gestation. USA percentiles were all higher than our data (18). The maximum differences between the 90^th^ percentile values were 286 g in the boys and 263 g in the girls for weight, 2 cm in both genders for length, as well as 1 cm in the boys and 1.5 cm in the girls for head circumference (Figures 2, 3).

**Figure 4:**
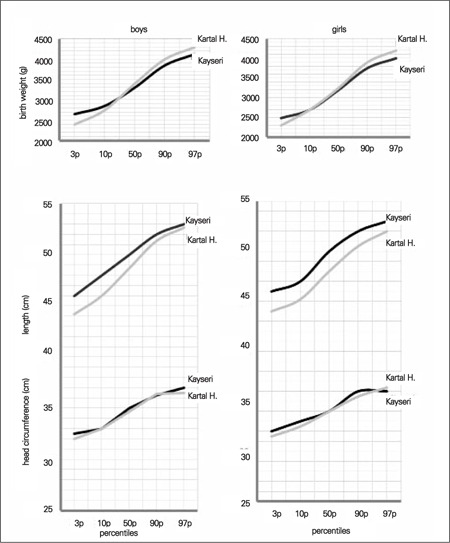
Comparison of 10th, 50th and 90th centiles of the Kayseri and Kartal Hospital samples for birth weight, birth length and head circumference at birth

We also compared graphically the 3^rd^, 10^th^, 50^th^, 90^th^ and 97^th^ percentiles obtained for our term infants with those reported in the Kartal Hospital study on term neonates. We found that birth weight in our term infants of both genders was approximately 200 g higher in the 3^rd^ and 10^th^ percentiles, but lower in the 90th and 97^th^ percentiles (17). There was no detectable difference between head circumferences in the two studies in either gender. The apparent difference between these two studies was in length at birth. The differences between the 3^rd^ and 10^th^ percentile values in the two studies were approximately 2 cm in both genders, but in the higher percentile groups, this difference decreased to 0.3 cm in boys and 1 cm in girls (Figure 4). 

## DISCUSSION

This study was designed to produce intrauterine growth percentiles. Weight, length and head circumference are regarded as the fundamental anthropometric measurements which are used by physicians (pediatricians, family physicians, gynecologists and obstetricians) in their clinical practice as well as by parents who desire to assess and follow the actual and future growth pattern of their siblings. Gestational age- and gender-specific anthropometric references also provide a basis for estimation of SGA and LGA prevalences and data for comparison with other studies. SGA infants have adverse outcomes other than growth problems such as neurodevelopmental delay (19,20). LGA infants are at risk for early hypoglycemia and metabolic syndrome in later years (21,22). Both preterm infants, especially those born younger than 32 weeks of gestation, and full-term SGA infants carry long-term health risks. These risks include adiposity and obesity (23,24). Intrauterine growth curves are the easiest and most reliable measure to predict these risks. All previous studies that were available to us were either conducted in a single hospital or did not report data for each gestational week or gender. 

We believe that the contribution of this study is to have produced gestational age-and gender-specific percentiles based on a population of neonates from several hospitals in a quite large city. To date, the 3^rd^, 5^th^, and 10^th^ birth weight percentiles have been used as cut-off values to identify clinically important fetal growth restriction (25). On the other hand, according to a consensus statement from an independent panel of pediatrics endocrinologists, SGA can be defined as a state of having a birth weight and/or length of at least 2 standard deviations (SD) below the mean for gestational age (26). The cut-off values for LGA, on the other hand, have been set at the 90^th^, 95^th^ or 97^th^ percentile by different investigators (27,28). This present study provides data for use of different criteria to define SGA. In our study, when the 10^th^ and 90^th^ percentiles were used as the cut-off values, the prevalences of SGA and LGA were found as 10.1% and 10.8%, respectively. 

There are several previous studies reporting gender difference for birth weight and length throughout pregnancy or at a certain gestational week (^17^,^18^,^19^,^29^). Our data also revealed that percentile values were higher in the males than in the females after the 35th week of gestation. The gender difference significantly increased in the 90^th^ percentile range for all anthropometric measurements.

Data from a global survey showed variations in birth weight among several countries. In this report, birth weight in term infants varied from 2790 g in India to 3511 g in Algeria (30,31). 

Comparing our data with those of USA newborns, we found that the percentile values of the neonates in our sample were somewhat higher before 32 weeks of gestation. After the first 32-35 weeks of gestation, USA percentiles were strikingly higher than the percentile values in our study (Figures 2 and 3). The potential contributory factors for this difference may be genetics and race, maternal size, maternal nutrition during pregnancy, smoking and other environmental determinants. 

Comparing our results with data from other national studies, such as the Kartal Hospital data (17), we found that our term infants had higher length but lower weight and similar head circumferences (Figure 4). This finding may be explained by socioeconomic differences, but it must be noted that the reported data were produced from the measurements of newborns in a single hospital. 

The potential limitations of this present study can be listed as the possibility of measurement errors in preterm neonates, the use of a cross-sectional method which limits the monitoring of the actual growth pattern, collection of data from different hospitals, the relatively small sample size, and the potential errors due to the reporting of the last menstrual period. However, this study provides a quite large sample size and detailed information. Both the percentile and the mean and standard deviation values we report can be used in comparisons with national or international data. We believe that these data will be of use both in clinical practice and for research purposes until more comprehensive, reliable and accessible national data pertaining to the intrauterine growth of Turkish infants are produced. 

## Figures and Tables

**Table 1:**
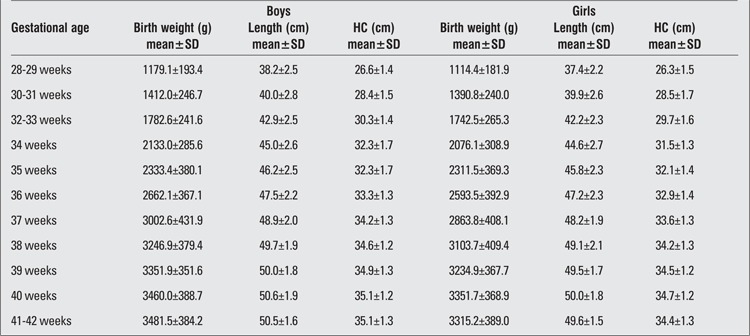
Gender-/gestational age-specific mean±standard deviation (SD) values for birth weight, length and head circumference (HC)

**Table 2:**
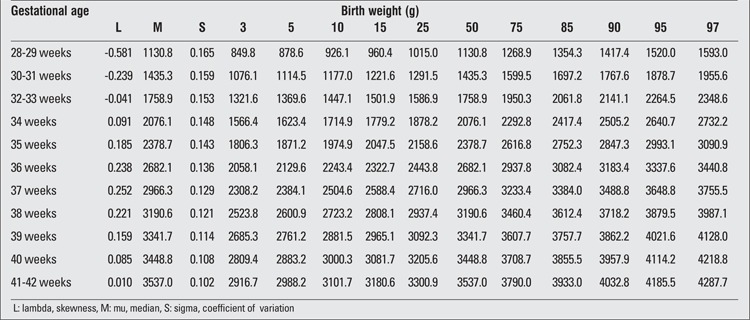
Birth weight percentiles by gestational age (boys)

**Table 3:**
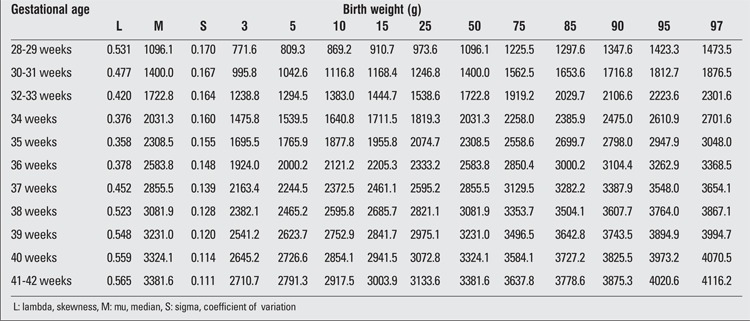
Birth weight percentiles by gestational age (girls)

**Table 4:**
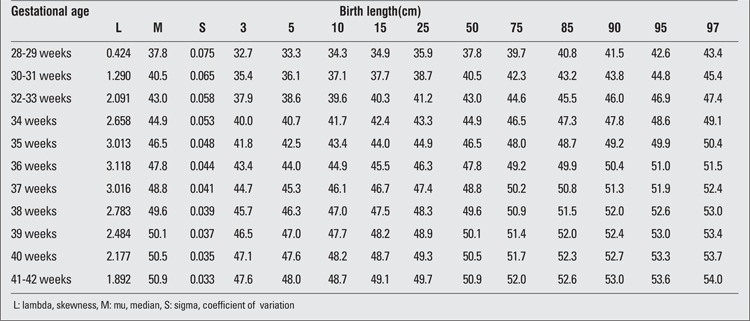
Birth length percentiles by gestational age (boys)

**Table 5:**
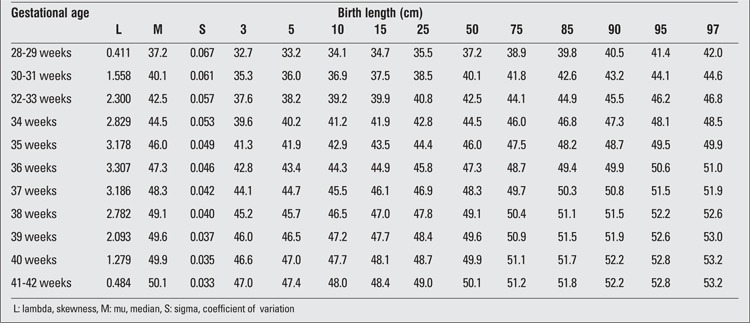
Birth length percentiles by gestational age (girls)

**Table 6:**
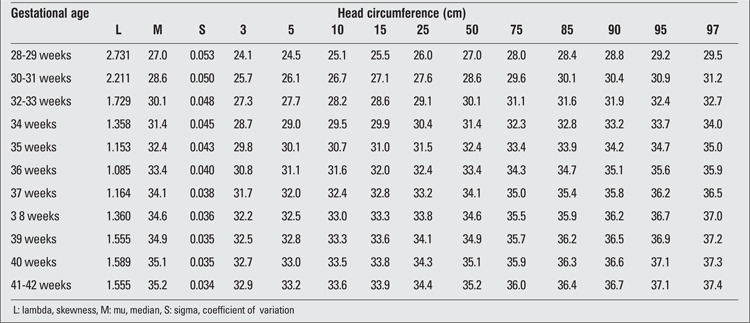
Head circumference percentiles at birth by gestational age (boys)

**Table 7:**
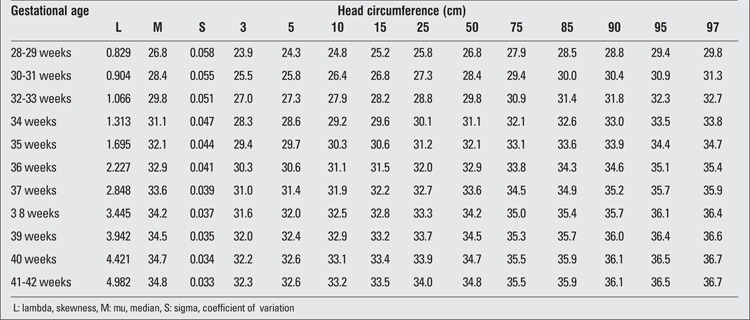
Head circumference percentiles at birth by gestational age (girls)

## References

[1] McCormick MC (1985). The contribution of low birth weight to infant mortality and childhood morbidity. N Engl J Med.

[2] Zhang X, Platt RW, Cnattingius S, Joseph KS, Kramer MS (2007). The use of customised versus population-based birthweight standards in predicting perinatal mortality. BJOG.

[3] McIntire DD, Bloom SL, Casey BM, Leveno KJ (1999). Birth weight in relation to morbidity and mortality among newborn infants. N Engl J Med.

[4] Meshari AA, De Silva S, Rahman I (1990). Fetal macrosomia-maternal risks and fetal outcome. Int J Gynaecol Obstet.

[5] Grassi AE, Giuliano MA (2000). The neonate with macrosomia. Clin Obstet Gynecol.

[6] Sparks JW, Ross JC, Cetin I, Polin AR, Fox WW (1998). Intrauterine growth and nutrition. Fetal and neonatal physiology.

[7] Lubchenco LO, Hansman C, Boyd E (1966). Intrauterine growth in length and head circumference as estimated from live births at gestational ages from 26 to 42 weeks. Pediatrics.

[8] Babson SG, Benda GI (1976). Growth graphs for the clinical assessment of infants of varying gestational age. J Pediatr.

[9] Curhan GC, Chertow GM, Willett WC, Spiegelman D, Colditz GA, Manson JE, Speizer FE, Stampfer MJ (1996). Birth weight and adult hypertension and obesity in women. Circulation.

[10] Tanner JM (1987). Growth as a mirror of the condition of society: secular trends and class distinctions. Acta Paediatr Jpn.

[11] Gardosi J Fetal growth standards: individual and global perspectives. Lancet.

[12] Gardosi J (1995). Ethnic differences in fetal growth. Ultrasound Obstet Gynecol.

[13] Neyzi O, Gunoz H, Celenk A, Bundak R (1986). Birth weight in Turkish infants. Hum Biol.

[14] Tumerdem Y, Ayhan B, Saygili H, Erbaydar E (1993). Metropolitan bir kent olan İstanbul’a intrauterine büyüme indeksleri. Çocuk Sağlığı ve Hastalıkları Dergisi.

[15] Yuksel B, Evliyaoglu N, Altintas D, Atici A, Alpaslan N, Serbest M, Yilmaz L (1996). Adana bölgesinde zamanında ve premature doğan bebeklerin ağırlık, boy, baş çevresi ölçümleri ve ponderal indeksleri. Çocuk Sağlığı ve Hastalıkları Dergisi.

[16] Ovali F (2003). Intrauterine growth curves for Turkish infants born between 25 and 42 weeks of gestation. J Trop Pediatr.

[17] Telatar B, Comert S, Vitrinel A, Erginoz E (2009). Anthropometric measurements of term neonates from a state hospital in Turkey. East Mediterr Health J.

[18] Olsen IE, Groveman SA, Lawson ML, Clark RH, Zemel BS (2010). New intrauterine growth curves based on United States data. Pediatrics.

[19] Hack M, Schluchter M, Cartar L, Rahman M, Cuttler L, Borawski E (2003). Growth of very low birth weight infants to age 20 years. Pediatrics.

[20] Hediger ML, Overpeck MD, Maurer KR, Kuczmarski RJ, McGlynn A, Davis WW (1998). Growth of infants and young children born small or large for gestational age: findings from the Third National Health and Nutrition Examination Survey. Arch Pediatr Adolesc Med.

[21] Hooy SK, Amorde-Spalding K, Groh-Wargo S, Thompson M, Cox JH, Hartline JV (2000). Infants of diabetic mothers. Nutritional Car efor High-Risk Newborns.

[22] Boney CM, Verma A, Tucker R, Vohr BR (2005). Metabolic syndrome in childhood: association with birth weight, maternal obesity, and gestational diabetes mellitus. Pediatrics.

[23] Finken MJ, Keijzer-Veen MG, Dekker FW, Frolich M, Hille ET, Romijn JA, Wit JM, Dutch POPS-19 Collaborative Study Group (2006). Preterm birth and later insulin resistance: effects of birth weight and postnatal growth in a population based longitudinal study from birth into adult life. Diabetologia.

[24] Euser AM, Finken MJ, Keijzer-Veen MG, Hille ET, Wit JM, Dekker FW, Dutch POPS-19 Collaborative Study Group (2005). Associations between prenatal and infancy weight gain and BMI, fat mass, and fat distribution in young adulthood: a prospective cohort study in males and females born very preterm. Am J Clin Nutr.

[25] Bhargava SK, Bhargava V, Kumari S, Madhavan S, Ghosh S (1974). Outcome of babies with severe intra-uterine growth retardation. I. Maternal factors, congenital malformations, mortality and survival pattern. Indian J Med Res.

[26] Lee PA, Chernausek SD, Hokken-Koelega AC, Czernichow P, International Small for Gestational Age Advisory Board (2003). International Small for Gestational Age Advisory Board consensus development conference statement: management of short children born small for gestational age, April 24-October 1, 2001. Pediatrics.

[27] Henriksen T (2008). The macrosomic fetus: a challenge in current obstetrics. Acta Obstet Gynecol Scand.

[28] Weissmann-Brenner A, Simchen MJ, Zilberberg E, Kalter A, Weisz B, Achiron R, Dulitzky M (2012). Maternal and neonatal outcomes of large for gestational age pregnancies. Acta Obstet Gynecol Scand.

[29] Thomas P, Peabody J, Turnier V, Clark RH (2000). A new look at intrauterine growth and the impact of race, altitude, and gender. Pediatrics.

[30] Mikolajczyk RT, Zhang J, Betran AP, Souza JP, Mori R, Gulmezoglu AM, Merialdi M (2011). A global reference for fetal-weight and birthweight percentiles. Lancet.

[31] Shah A, Faundes A, Machoki M, Bataglia V, Amokrane F, Donner A, Mugerwa K, Carroli G, Fawole B, Langer A, Wolomby JJ, Naravaez A, Nafiou I, Kublickas M, Valladares E, Velasco A, Zavaleta N, Neves I, Villar J (2008). Methodological considerations in implementing the WHO Global Survey for Monitoring Maternal and Perinatal Health. Bull World Health Organ.

